# P-616. Sociodemographic and Clinical Factors Affecting Vaccination after Pediatric Solid Organ Transplantation at Duke University

**DOI:** 10.1093/ofid/ofae631.814

**Published:** 2025-01-29

**Authors:** Adam Blatt, Yeh-Chung Chang, Yunfei Wang, Sarah M Heston

**Affiliations:** Duke University, Durham, North Carolina; University of Texas Southwestern Medical Center, Dallas, Texas; Duke Human Vaccine Institute, Durham, North Carolina; Duke University, Durham, North Carolina

## Abstract

**Background:**

Pediatric solid organ transplant (SOT) recipients are at increased risk for vaccine preventable illnesses (VPI). They frequently receive vaccines after transplantation when immunosuppressive medications and cytopenias may impact their immune response to routine childhood vaccines. Current guidelines recommend monitoring vaccine-specific serologies to ensure optimal vaccine responses and to guide the need for repeat doses.

**Figure:**
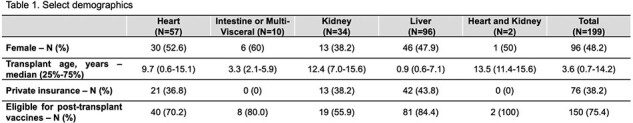

**Methods:**

In this single-center retrospective cohort study, a multivariate logistic regression model was used to determine the sociodemographic and clinical factors associated with delays in starting post-transplant vaccines and adherence to post-vaccine serologic monitoring among 199 pediatric patients who received a SOT at Duke University between 2014-2023. The prevalence of cytopenias at the time of post-transplant vaccination was also described.

**Figure:**
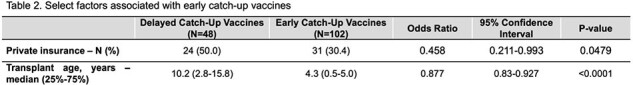

**Results:**

Out of 150 children eligible for post-transplant vaccines (Table1), 48 (32%) failed to start catch-up vaccines within 2 years of transplantation. Older children (OR=0.877, 0.83-0.927) and children with private insurance (OR=0.458, 0.211-0.993) had significantly decreased odds of early catch-up vaccines (Table 2). Hepatitis B serologies were obtained in 23% of patients after post-transplant vaccination, but titers against the remaining non-live childhood vaccines were only obtained in 1-8% of children (Table 3). Approximately 53% of all children had neutropenia or lymphopenia (less than 1500 x 10^6 cells/L) at the time of post-transplant vaccination, with heart and intestinal/multi-visceral transplant recipients having the highest rates at ∼75% (Table 4).

**Figure:**
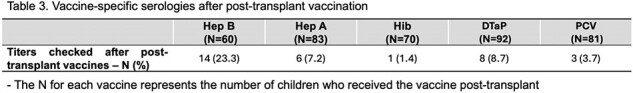

**Conclusion:**

These findings identify pediatric SOT recipients most at risk for delayed post-transplant vaccines. The associations between vaccine delay and private insurance and older age warrant further investigation. The extremely low rates of vaccine-specific serologic monitoring and the high prevalence of cytopenias at the time of vaccination raise concerns that pediatric SOT recipients are insufficiently protected from VPI. Future work will determine the impact of cytopenias on vaccine response and will address inequities in vaccination among pediatric SOT recipients.

**Figure:**
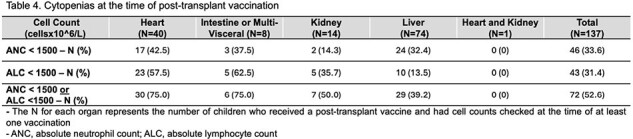

**Disclosures:**

**All Authors**: No reported disclosures

